# Evaluation of the human placenta optical scattering properties using continuous wave and frequency-domain diffuse reflectance spectroscopy

**DOI:** 10.1117/1.JBO.25.11.116001

**Published:** 2020-11-05

**Authors:** Siddharth M. Khare, Thien Nguyen, Afrouz A. Anderson, Brian Hill, Roberto Romero, Amir H. Gandjbakhche

**Affiliations:** a National Institute of Child Health and Human Development, National Institutes of Health, Bethesda, Maryland, United States; b U.S. Department of Health and Human Services, Eunice Kennedy Shriver National Institute of Child Health and Human Development, National Institutes of Health, Perinatology Research Branch, Division of Obstetrics and Maternal-Fetal Medicine, Division of Intramural Research, Bethesda, Maryland, and Detroit, Michigan, United States; c University of Michigan, Department of Obstetrics and Gynecology, Ann Arbor, Michigan, United States; d Michigan State University, Department of Epidemiology and Biostatistics, East Lansing, Michigan, United States; e Wayne State University, Center for Molecular Medicine and Genetics, Detroit, Michigan, United States; f Detroit Medical Center, Detroit, Michigan, United States; g Florida International University, Department of Obstetrics and Gynecology, Miami, Florida, United States

**Keywords:** diffuse optical spectroscopic system, near-infrared spectroscopy, power function, random walk theory, scattering coefficient, tissue oxygenation

## Abstract

**Significance:** Placenta is an essential organ for fetal development and successful reproduction. Placental insufficiency can lead to fetal hypoxia and, in extreme cases anoxia, leading to fetal death. Of the 145 million deliveries per year worldwide, ∼15 million neonates are small for gestational age and, therefore, at risk for antepartum and intrapartum hypoxia. Clinical methods to assess placental function largely rely on the assessment of fetal heart rate changes but do not assess placental oxygenation. Near-infrared spectroscopy (NIRS) allows non-invasive, real-time assessment of tissue oxygenation in intact organs, which can be used to assess placental oxygenation. However, tissue optical properties can affect the accuracy of methods to measure tissue oxygenation.

**Aim:** This study was performed to estimate the scattering coefficient of the human placenta. We have computed the scattering coefficients of the human placenta for the range of 659 to 840 nm using two methods of diffuse reflectance spectroscopy (DRS).

**Approach:** Measurements were performed using an in-house DRS device and a well-established frequency-domain diffuse optical spectroscopic system (DOSI). Measurements were performed in eight placentas obtained after cesarean deliveries. Placentas were perfused with normal saline to minimize the effects of absorption due to blood. Three sites per placenta were measured. Absorption and scattering coefficients were then calculated from the measured reflectance using the random walk theory for DRS and frequency-domain algorithm for DOSI.

**Results:** Average reduced scattering coefficient (${\mu s}^{\prime }$) was $0.943\pm 0.015\;{\mathrm{mm}}^{-1}$ at 760 nm and $0.831\pm 0.009\;{\mathrm{mm}}^{-1}$ at 840 nm, and a power function ${\mu s}^{\prime }=1.6619$ ${\left(\lambda /500\;\mathrm{nm}\right)}^{-1.426}$ was derived for the human placental scattering coefficient.

**Conclusion:** We report for the first time the scattering coefficient of the human placenta. This information can be used to assess baseline scattering and improve measurements of placental oxygen saturation with NIRS.

## 1 Introduction

The placenta is an essential organ for fetal development and successful pregnancy.^1^ The villous tree of this organ provides an interface between the maternal and fetal circulations.^1^ The respiratory function of the placenta consists of providing oxygen from maternal blood to the fetus, and carbon dioxide from the fetus into the maternal circulation.^2^ Other important functions of the placenta include providing nutrients to maintain fetal growth, waste removal, endocrine, and immunological functions.^1^

The term “placental insufficiency” is used in clinical obstetrics to refer to a state in which the organ fails to deliver nutrients or oxygen, which may lead to fetal growth restriction or, in extreme cases, death from anoxia.^3^^–^^6^ Most tests of placental function rely on indirect parameters, such as the assessment of fetal growth, Doppler assessment of vascular impedance in the maternal or fetal circulation, or the determination of placental products such as human chorionic gonadotropin, human placental lactogen, placental-specific protein 13, or other products manufactured by the organ.^7^ Given the importance of placental respiratory function for fetal survival, a method to assess placental oxygenation in real time would be of considerable importance in clinical medicine, and in the understanding of the pathophysiology of pregnancy.

Tissue oxygenation can be measured *in-vivo* by calculating the tissue oxygenation index (TOI). Blood oxygen saturation is now routinely used in clinical medicine to assess respiratory function and is part of routine practice. The most common method to assess oxygenation is using optical methodologies, which largely rely on diffuse reflectance spectroscopy (DRS).^8^^–^^10^ Typically, for this purpose, light in the near-infrared (NIR) range (700 to 900 nm) of wavelengths is directed to a tissue in close proximity to the circulation. Given that NIR can penetrate tissue without substantial attenuation by normal components, such as adipose tissue and water, light can diffuse into the tissue from a source and backscattered light can be detected with optical methods. The principle behind this technology consists of the detection of changes in the concentrations of oxyhemoglobin (HbO) and deoxyhemoglobin.

To reach the placenta *in-vivo*, the light needs to pass through the skin, adipose tissue, and myometrium (uterus). In another study on 12 pregnant women in the third trimester, we found the total thickness of these tissues ranged from 7.1 to 42.5 mm (unpublished data). Near-infrared spectroscopy (NIRS) can probe depths of up to 30-mm below the skin surface. This was determined using Monte Carlo photon diffusion simulations of a three-layer tissue (skin, fat, and muscle) using the hop-drop-spin method described by Steve Jacques (data not yet published). Therefore, we propose that NIRS will be able to noninvasively probe the upper few millimeters of the placenta in a pregnant woman with total maternal tissue thickness <30-mm and with the placenta positioned in the anterior, fundal, and side of the uterus.

There have been very few studies on placental oxygenation.^11^^–^^13^ Matsuo et al.^11^ reported that placental oxygenation is lower in preeclampsia, measured by the umbilical arterial-venous oxygen difference. Hasegawa et al. used an NIRS device to perform transabdominal measurements of placental TOI in pregnancies complicated with a small for gestational age fetus. They reported higher placental TOI values for fetuses whose mothers had preeclampsia and placental abnormalities, whereas those with umbilical cord abnormalities showed a lower placental TOI values.^12^ Moreover, the authors proposed that a high TOI represented a greater than normal concentration of HbO, which resulted from a reduced oxygen exchange caused by placental pathology in the intervillous space. Kakogawa et al.^13^ also reported that the NIRS probe can be utilized to assess placental oxygenation. However, these studies have not taken into account sufficient information about the optical scattering of the placenta.

Because both scattering and absorption contribute to the light attenuation in the tissue and the soft tissue is in general a highly scattering medium, incorporating scattering coefficient of placental tissue can improve the reliability of the assessment of tissue oxygenation. Currently, the scattering coefficient of the human placental tissue is unknown. Herein, we report the effective/reduced scattering coefficient (${\mu s}^{\prime }$) in *ex-vivo* placental tissue samples.

## 2 Materials and Methods

### 2.1 Measurement Devices

We chose two devices to measure placental optical properties: (1) an in-house continuous-wave (CW) system, featuring two wavelengths. This device records spatially resolved diffused reflectance with source-detector (SD) separation from 7.4 to 54.4 mm and (2) a frequency-domain diffuse optical spectroscopic system (DOSI) with a fixed SD separation of 28 mm.

#### 2.1.1 Continuous-wave spatially diffuse reflectance spectroscopy

**Fig. 1:**
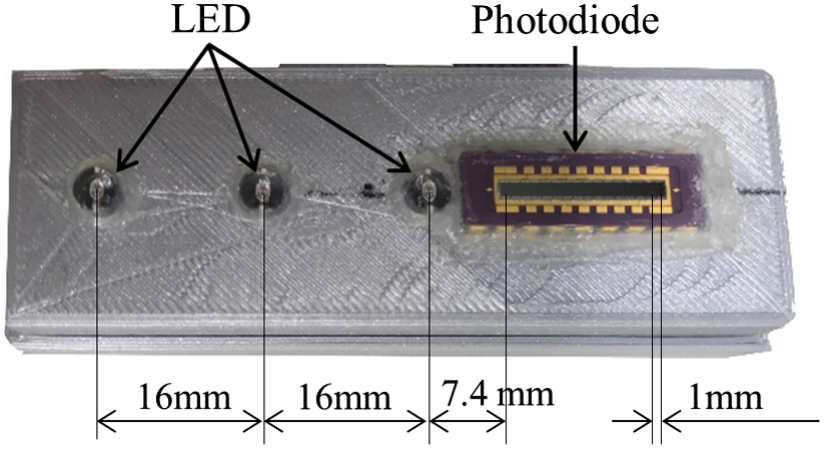
DRS device built in-house for measurement of the optical properties of the placental tissue.

Figure 1 shows the in-house DRS device, which consists of a 16-element photodiode array (S4111-16R with driver circuit C9004, Hamamatsu Photonics K.K., Hamamatsu City, Shizuoka, Japan) as a detector and three dual-wavelength NIR light-emitting diodes (LEDs; L760/840-05A, Ushio Inc., Tokyo, Japan) as the light sources. The center-to-center distance between consecutive elements in the photodiode array is 1 mm. The LEDs emit light at 760 and 840 nm and are placed at distances of 7.4, 23.4, and 39.4 mm from the first element of the photodiode array. The system was custom-built for high-speed semi-automated data acquisition and storage. Data were acquired with 10-bit resolution. A frame to hold the light sources and detector was 3D-printed to make the assembly easy to handle. It was sealed with silicone glue to prevent moisture from entering the device. The system can measure a diffuse reflectance profile with 1-mm spatial resolution in the SD distance range from 7.4 to 54.4 mm. The LEDs were designed to emit light at four different intensity levels, each measured with a power meter (S120C, Thorlabs, New Jersey) to compensate for the LED-to-LED variations. The detection performance of all 16 photodiode elements was measured. The differences among the elements were negligible and have not been accounted for herein.

#### 2.1.2 Frequency-domain diffuse optical spectroscopic system measurements

The frequency-domain system, also called the DOSI, is well-characterized and has been previously described.^14^^,^^15^ Measurements were performed using a hand-held probe with a fixed SD distance of 28 mm. Amplitude-modulated near-infrared light at four wavelengths (659, 689, 781, and 829 nm) were utilized for frequency-domain measurements; the broadband continuous wave mode of the device was not enabled. The modulated frequency of DOSI ranges from 40 to 400 MHz, with a source power of all four wavelengths of 20 mW. The detector is an avalanche photodiode with 1.5-mm diameter (Hamamatsu Photonics K.K., Hamamatsu City, Shizuoka, Japan). Details of the system calibration and algorithms used in this system to measure absorption and reduced scattering coefficients have been reported.^14^^–^^16^

### 2.2 Placental Tissue Preparation

**Fig. 2:**
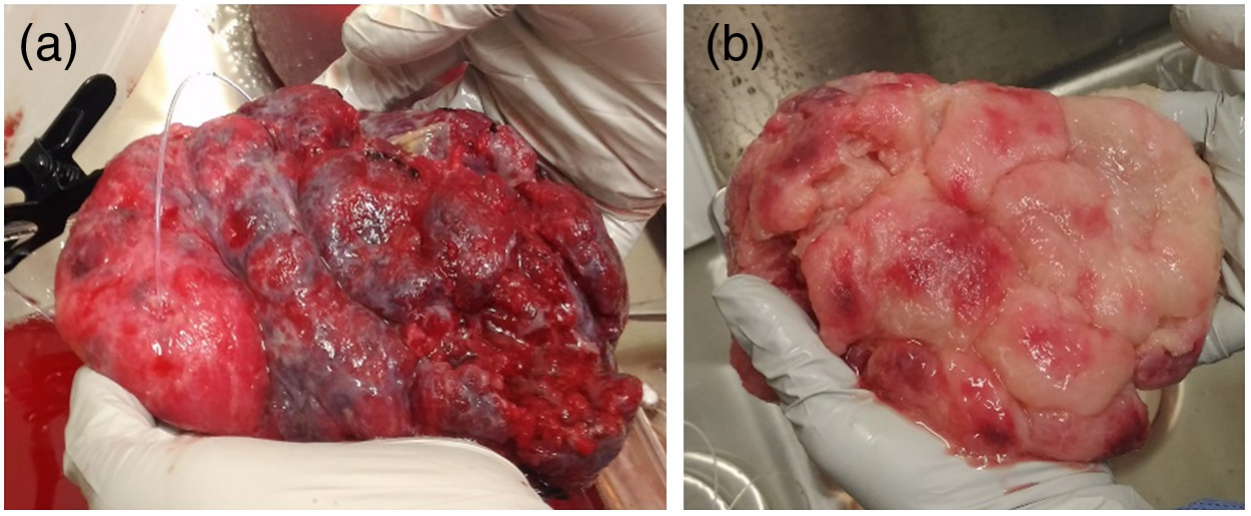
Placental tissue (a) during perfusion of the maternal lobe. Removal of blood can be observed in the lighter color of the lobe on the left, and (b) after perfusion of all maternal lobes.

Eight normal placentas (gestational age: 37 to 41 weeks) were obtained from cesarean deliveries with pregnancies without complication at Walter Reed Hospital (Bethesda, Maryland). The maternal age was between 18 and 40 years old. This study excluded all placentas from mother with major fetal congenital anomalies, maternal hypertension/hypertensive disorder, diagnosis of intraamniotic infection, diagnosis of intrauterine growth restriction, maternal diabetes, hypertension, multiple gestation, and placental abnormalities. In addition, placentas that required pathological analysis were excluded. The approximate thickness of the placental tissues was 25 mm. All placentas were delivered to the laboratory within 30 min of the procedure. The whole placentas were perfused with saline to remove the blood for accurate assessment of the reduced scattering coefficient. The time-lapse between receiving the placenta and the onset of perfusion was about 6 min. The umbilical vein was cannulated, and the placenta was perfused with a phosphate-buffered saline 1× solution using a peristaltic pump at the flow rate of 100  ml/min for about 30 min. After perfusion of the fetal side, the maternal side was perfused for about 45 to 60 min by inserting thin Teflon tubes at multiple locations in the maternal lobes. The flow rate from each tube was ∼10  ml/min. The DRS device was utilized to measure optical properties at multiple placental locations. This study was approved by the National Institutes of Health and the Walter Reed National Military Medical Center (Bethesda, Maryland). Figure 2 shows the maternal side of the placenta before and after perfusion, respectively.

### 2.3 Estimation of Reduced Scattering Coefficient (μs′) and Absorption Coefficient (μa)

The random walk theory for diffusion of photons through an absorbing and scattering medium has been previously described.^17^ For a semi-infinite medium, the probability that a photon is diffusely reflected to the surface of the medium at a distance r from the source is given by Eq. (1), and μa and $\mu s^{\prime }$ are the absorption and reduced scattering coefficients, respectively.

$$\Gamma (r)=\;\frac{e^{-2\frac{\mu_{a}}{\mu_{s}^{\prime }}}}{4\pi \frac{\mu_{s}^{\prime }r}{\sqrt{2}}}\;[e^{-r\sqrt{3\mu_{a}\mu_{s}^{\prime }}}-e^{\left(-\sqrt{6\frac{\mu_{a}}{\mu_{s}^{\prime }}(\frac{\mu_{s}^{\prime 2}r^{2}}{2}+4)}\right)}].$$ (1)

The intensity of light detected at the surface depends on the photon flux that, in turn, depends on the characteristics of the light source and detection efficiency. In our device, the intensity is measured in terms of the photodiode count (range: 0 to 1023). As a result, the intensity values are expressed in arbitrary units. To account for the source intensity, we calculated the intensity I(r) at distance r from the source as

I(r)=αΓ(r). (2)

The constant α is the instrument calibration factor that corresponds to the source intensity. To determine α, we employed a reference phantom made of Polydimethylsiloxane (PDMS), TiO2 powder, and India ink. The optical properties μa and $\mu s^{\prime }$ of this phantom were determined using a well-characterized frequency-domain system (DOSI at Beckman laser institute, University of California, Irvine in Irvine, California) and reconfirmed by a commercial time-domain system (Hamamatsu Photonics K.K., Hamamatsu City, Shizuoka, Japan), $\mu a=0.0052\pm 0.0001\;{\mathrm{mm}}^{-1}$ and ${\mu s}^{\prime }=0.6914\pm 0.0083\;{\mathrm{mm}}^{-1}$ at 760 nm and $\mu a=0.0036\pm 0.0001\;{\mathrm{mm}}^{-1}$ and ${\mu s}^{\prime }=0.6148\pm 0.0095\;{\mathrm{mm}}^{-1}$ at 840 nm. These values were substituted in the random walk Eq. (1) to obtain the probability of the diffuse reflectance Γ(r). Next, the in-house DRS device was used on the reference phantom to record the reflection profile I(r) at all possible SD distances r. From Eq. (2), the instrument calibration factor α was calculated using the measured intensity values and the derived Γ(r) values.

Equation (2), with the known α at 760 and 840 nm, was then applied to calculate the optical properties using a least-square fit to the data measured by the DRS device. The least-square fitting was performed utilizing the Matlab^®^ software (The MathWorks^®^ Inc, Massachusetts) Curve Fitting Tool (95% confidence bounds). The first 16 SD separations were included in our analysis as the larger SD separation yielded insufficient intensities. This methodology was validated using three phantoms, whose optical properties at four different wavelengths were provided by either the company (fNIR Devices LLC, New Orleans, Louisiana, 680, 750, 780, and 810 nm) or measured with a DOSI system and was confirmed with the time domain system measurement. Since the provided wavelengths were different with the wavelengths used in our DRS device, we could only compute ${\mu s}^{\prime }$ (not μa) at 760 and 840 nm by a power function fitting (Table 1). The ${\mu s}^{\prime }$ values obtained using the DRS devices have an average of 7.2% deviation from the provided/FD-measured values.

**Table 1:** Scattering coefficients of the three phantoms used for validation.

Phantom name	Source of ${\mu s}^{\prime }$ values	${\mu s}^{\prime }$ at 760 nm (${\mathrm{mm}}^{-1}$)		${\mu s}^{\prime }$ at 840 nm (${\mathrm{mm}}^{-1}$)	
		Standard	DRS	Standard	DRS
fNIR devices LLC	Provided by the company	1.06	0.93	0.96	0.85
Acrin7	Measured using DOSI	0.66	0.70	0.57	0.60
Scrooge	Measured using DOSI	0.91	0.88	0.82	0.78

The above method was then applied to the DRS intensity data from the perfused placentas to obtain their optical properties.

## 3 Results

### 3.1 Random Walk Theory Fitting

**Fig. 3:**
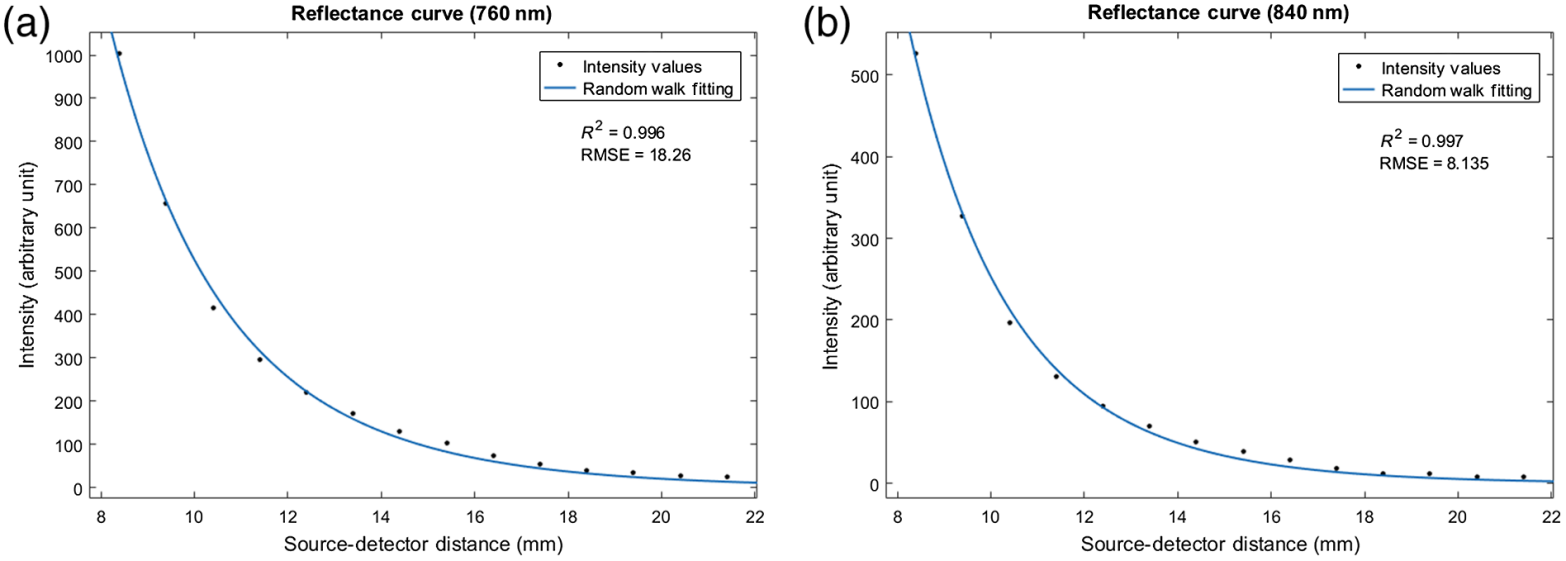
Diffuse reflectance profiles at the maternal side of a perfused placenta: Measured intensity values (black dots) plotted as a function of SD distance and random walk fit (blue solid line) at (a) 760 and (b) 840 nm.

The diffuse reflectance profile was measured on the maternal side of the perfused placenta. The small amount of blood that remains after perfusion within the tissue is expected to absorb the photons without changing the optical scattering of the tissue significantly. Figure 3 shows representative diffuse reflectance profiles measured utilizing the in-house DRS system on the maternal side of a perfused placenta. The random walk model fits well to the DRS profiles. The goodness of the fit was as follows: $R^{2}=0.996$ and RMSE=18.26 at 760 nm and $R^{2}=0.997$ and RMSE=8.135 at 840 nm. The instrument calibration factors, calculated as described in Sec. 2.3, were 1140261 at 760 nm and 902105 at 840 nm.

### 3.2 Absorption and Reduced Scattering Coefficients Derived from the CW DRS Device

For each placenta, the measurements and calculations were performed at three sites on the maternal side with each region being measured three times. The reduced scattering (${\mu s}^{\prime }$) and absorption (μa) coefficients of a placenta were then obtained by averaging data from nine measurements for each placenta. Data from one of eight placentas were excluded because of the low measured intensity. Reduced scattering (${\mu s}^{\prime }$) and absorption (μa) coefficients of seven perfused placental tissues are given in Table 2. The mean reduced scattering and absorption coefficients of perfused placental tissue are ${\mu s}^{\prime }=0.943\pm 0.015\;({\mathrm{mm}}^{-1})$ and $\mu a=0.012\pm 0.002\;({\mathrm{mm}}^{-1})$ at 760 nm and ${\mu s}^{\prime }=0.831\pm 0.009\;({\mathrm{mm}}^{-1})$ and $\mu a=0.007\pm 0.001\;({\mathrm{mm}}^{-1})$ at 840 nm. The ${\mu s}^{\prime }$ values did not vary drastically despite the observed changes in the μa values (Table 2).

**Table 2:** Mean and standard errors of reduced scattering (${\mu s}^{\prime }$) and absorption (μa) coefficients of seven perfused placental tissues.

Placenta	${\mu s}^{\prime }$ (760) (${\mathrm{mm}}^{-1}$)	μa (760) (${\mathrm{mm}}^{-1}$)	${\mu s}^{\prime }$ (840) (${\mathrm{mm}}^{-1}$)	μa (840) (${\mathrm{mm}}^{-1}$)
1	0.924 (±0.014)	0.005 (±0.003)	0.798 (±0.011)	0.006 (±0.003)
2	0.822 (±0.004)	0.008 (±0.0001)	0.721 (±0.004)	0.005 (±0.0001)
3	1.055 (±0.020)	0.018 (±0.002)	0.928 (±0.009)	0.013 (±0.0009)
4	1.020 (±0.012)	0.015 (±0.001)	0.903 (±0.009)	0.006 (±0.0005)
5	0.900 (±0.023)	0.015 (±0.004)	0.787 (±0.011)	0.007 (±0.002)
6	0.899 (±0.013)	0.008 (±0.0009)	0.821 (±0.007)	0.004 (±0.0004)
7	0.979 (±0.021)	0.017 (±0.001)	0.859 (±0.010)	0.005 (±0.001)
Mean	0.943 (±0.015)	0.012 (±0.002)	0.831 (±0.009)	0.007 (±0.001)

### 3.3 Reduced Scattering Coefficients Measured with the Frequency-Domain System

**Fig. 4:**
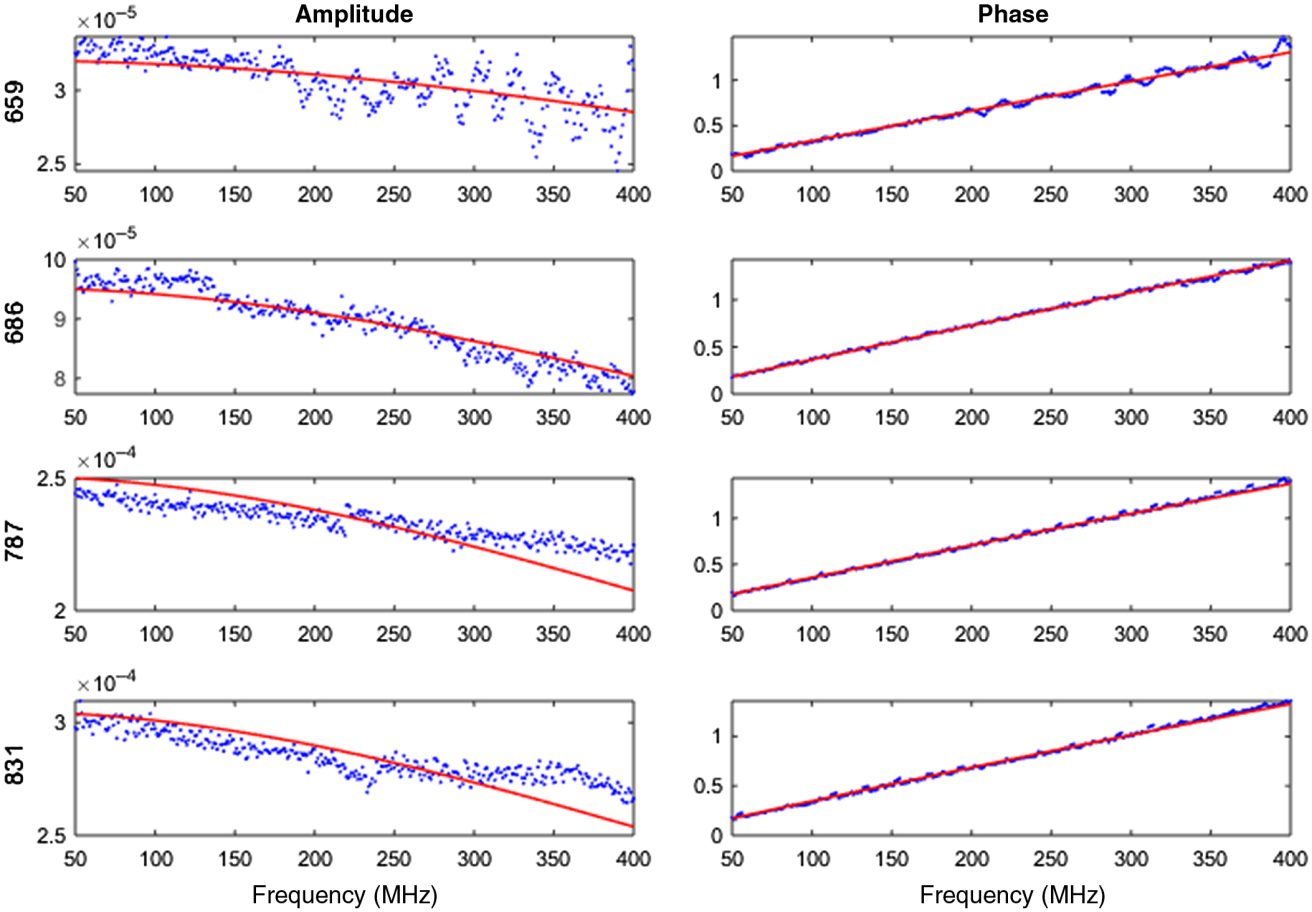
Amplitude and phase model fit of DOSI data measured on placenta 1

**Fig. 5:**
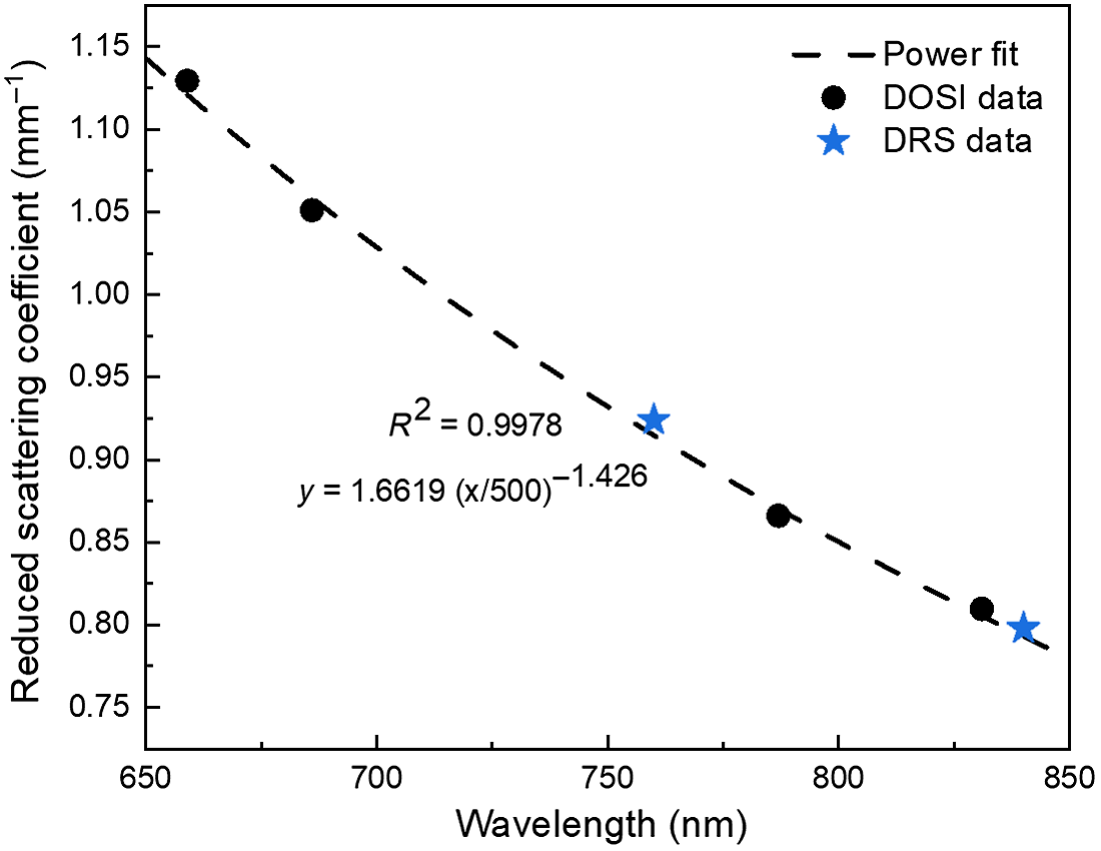
Reduced scattering coefficients calculated from two devices. The black dashed line shows the power fit of the DOSI measurement (black dots) while the blue dots represent the DRS measurements from placenta 1. Abbreviations: DOSI, diffuse optical spectroscopic system; DRS, diffuse reflectance spectroscopy.

The DOSI system provided optical property measurements at four wavelengths, hence it was used to measure the reduced scattering coefficients of one of the placentas (placenta 1, Table 2) and compared to the coefficients estimated with the DRS device. Figure 4 shows amplitude and phase model fits to the measured data on placenta 1 using the DOSI system. The measurement using the DOSI system yielded ${\mu s}^{\prime }=1.129\pm 0.025\;{\mathrm{mm}}^{-1}$ at 659 nm, ${\mu s}^{\prime }=1.050\pm 0.021\;{\mathrm{mm}}^{-1}$ at 686 nm, ${\mu s}^{\prime }=0.866\pm 0.019\;{\mathrm{mm}}^{-1}$ at 787 nm, and ${\mu s}^{\prime }=0.810\pm 0.015\;{\mathrm{mm}}^{-1}$ at 831 nm. A power function $\mu_{s}^{\prime }=a{\left(\lambda /500\;\mathrm{nm}\right)}^{-b}$ was fitted to these reduced scattering coefficients with a=1.6619 and b=1.426 with $R^{2}=0.9978$ (Fig. 5). The reduced scattering coefficients at 760 and 840 nm were then derived as ${\mu s}^{\prime }=0.915$ and $0.793\;{\mathrm{mm}}^{-1}$, respectively.

## 4 Discussion

In this study, we have shown the feasibility of using a continuous wave DRS system in assessment of reduced scattering coefficient of the placental tissue *ex-vivo*. However, it is important to emphasize that the measurement in this study was performed on well-perfused placenta tissues. Based on the study by Yao et al.,^18^ the placental blood volume fraction, when the umbilical cord was clamped 5 s after delivery was ∼21%. The removal of the blood from these placentas affects the measured reduced scattering coefficient from its value *in-vivo*. However, the residual blood volume varies by large amounts in the *ex-vivo* placentas. Placental blood is lost during the cesarean delivery, transportation of the placenta and the umbilical cord clamping time. According to Yao et al.,^18^ clamping time can change the residual placental blood volume up to 60%. In addition, even though the umbilical cord was clamped, the blood inside of the placenta still drained out from the maternal side during the transportation to the lab. Therefore, we report the reduced scattering coefficient of the underlying placental tissue without blood. Scattering properties of the blood will need to be considered when performing noninvasive monitoring of placental oxygenation with known or estimated placental blood volume fraction.

Additionally, the results from the in-house DRS device can be affected to some extent by the light reflected back into the tissue from the boundary between the tissue surface and the device. However, the difference in the DRS intensity was negligible (<1%) when measurements were performed on the Acrin phantom with and without coating the surface of the device using a black tape. Hence, we expect the effect of reflected light on calculated scattering coefficient is insignificant. In addition to reporting the optical scattering of the human placenta, this work provides a simpler, more compact, and cost-effective system and methodology to measure the scattering properties of the human placental tissue *ex-vivo*. These factors will help in generating a large database from normal and abnormal pregnancies. The data presented here act as a reference point for future studies and will help provide more accurate distribution of the optical scattering properties of the human placenta.

## 5 Conclusion

In this study, we used DRS in continuous wave and frequency-domain mode to measure the optical scattering coefficient of the human placenta without blood in the NIR range. The provided power law equation can be used to calculate the reduced scattering coefficient at any wavelength in the NIR band. This platform can facilitate the study of key parameters such as tissue oxygenation and blood flow within the placenta. The results reported herein could allow a more precise calculation of placental oxygenation and assess the respiratory function of the organ noninvasively. In the future, it would be important to assess the scattering properties of placentas obtained from normal pregnancy and those with complications. Overall, our in-house DRS device provides a simpler and compact means for measurement of optical properties on the placenta, which can help generate larger database.
